# Anthropometry measures and prevalence of obesity in the urban adult population of Cameroon: an update from the Cameroon Burden of Diabetes Baseline Survey

**DOI:** 10.1186/1471-2458-6-228

**Published:** 2006-09-13

**Authors:** Raoul M Kamadjeu, Richard Edwards, Joseph S Atanga, Emmanuel C Kiawi, Nigel Unwin, Jean-Claude Mbanya

**Affiliations:** 1 Health of Population in Transition (HoPiT) Research Group. Faculty of Medicine and Biomedical Sciences (FMSB). Yaoundé University I, Cameroon; 2 Department of Public Health, Wellington School of Medicine and Health Sciences, University of Otago, Wellington, New Zealand; 3 The Medical School, University of Newcastle-upon-Tyne. Department of Medicine and Human Diabetes and Metabolism Research Centre, the Medical School. University of Newcastle-upon-Tyne, UK

## Abstract

**Background:**

The objective of the study was to provide baseline and reference data on the prevalence and distribution of overweight and obesity, using different anthropometric measurements in adult urban populations in Cameroon.

**Methods:**

The Cameroon Burden of Diabetes Baseline Survey was a cross-sectional study, conducted in 4 urban districts (Yaoundé, Douala, Garoua and Bamenda) of Cameroon, using the WHO Step approach for population-based assessment of cardiovascular risk factors. Body mass index, waist circumference and waist-to-hip ratio were measured using standardized methods. Overall, 10,011 individuals, 6,004 women and 4,007 men, from 4,189 households, aged 15 years and above participated.

**Results:**

Based on body mass index, more than 25% of urban men and almost half of urban women were either overweight or obese with 6.5% of men and 19.5% of women being obese. The prevalence of obesity showed considerable variation with age in both genders. Using body mass index provided the highest prevalence of obesity in men (6.5%) and waist-to-hip ratio the lowest prevalence (3.2%). Among women, using waist-to-hip ratio and waist circumference yielded the highest prevalence of obesity (28%) and body mass index the lowest (19.5%). There was a trend towards an increase in age-adjusted odd ratios of being overweight or obese with duration of education in both sexes.

**Conclusion:**

The study provides current data on anthropometric measurements and obesity in urban Cameroonian populations, and found high prevalences of overweight and obesity particularly over 35 years of age, and among women. Prevalence varied according to the measure used. Our findings highlight the need to carry out further studies in Cameroonian and other Sub-Saharan African populations to provide appropriate cut-off points for the identification of people at risk of obesity-related disorders, and indicate the need to implement interventions to reverse increasing levels of obesity.

## Background

The prevalence of overweight and obesity is rapidly increasing in developing as well as industrialised countries [1-3]. A previous Cameroonian study in a rural population found very little gain in weight with age and very low prevalence of overweight and obesity [4]. However, studies from urban populations in Cameroon have shown that overweight and obesity are increasingly common [4,5]. For example, the estimated prevalence of obesity, based on BMI, was 17.1% in women and 5.4% in men in urban Cameroon in 2002 [4].

The evaluation of fatty mass and definitions of overweight and obesity use a range of approaches, some of which are complex or invasive, and are inapplicable outside of specialised clinical practice to identify candidates for weight management [6-8]. In routine clinical practice and epidemiological studies the most commonly used measure to define overweight and obesity is the Body Mass Index (BMI). In addition, central obesity is measured by increase in waist circumference (WC) or waist-to-hip ratio (WHR) [9]. Increased weight and waist circumference have been shown to be strongly associated with cardiovascular disease risk factors such as diabetes and hypertension in many populations [10-12].

The objective of the Cameroon Burden of Diabetes (CamBoD) Project was to carry out an extensive survey in four urban areas of Cameroon, using the WHO STEPS approach [13] to assess the prevalence of cardiovascular risk factors. The present paper aims to provide baseline and reference data on the prevalence and distribution of overweight and obesity, using anthropometric measurements in the adult population from four urban centres in Cameroon.

## Methods

### Study population

The methods used for this study have been described elsewhere [14]. The Cameroon Burden of Diabetes (CamBoD) was a cross-sectional study conducted in 4 urban districts (Yaoundé, Douala, Garoua and Bamenda) of Cameroon. The study used multilevel systematic sampling stratified by age group. A population of 23,000 males and females' adults aged 15 years and above were identified in the census from the study sites, and 10,824 individuals were invited to participate. Ten thousand eleven individuals (6,004 women and 4,007 men) from 4,189 households (range 1–20, mean of three participants per household) participated, giving an overall response rate of 92.5%. Each study site contributed approximately 25% of the total sample. The response rate was higher in females and in the lower age groups (< 35 years). The WHO STEPS approach (modified STEP Version 1.3 instrument [15]) for collecting surveillance data for non-communicable diseases was used to collect prevalence data for diabetes, hypertension and their risk factors.

### Measurements

Anthropometric measurements included weight, height, waist and hip circumference. Body weight in light clothes was measured to the nearest 0.1 kg using a Soehnle scale (Soehnle-Waagen GmbH & Co. KG, Wilhelm-Soehnle-Straße 2, D-71540 Murrhardt/Germany) and height to the nearest 0.5 cm using a portable locally manufactured stadiometers. Subjects stood upright on a flat surface without shoes, with the back of the heels and the occiput on the stadiometer. BMI was identified as weight divided by height squared (kg/m^2^). Four categories of BMI (≤20, 20–24.9, 25–29.9, and ≥30 kg/m^2^) were identified. The categories were selected according to WHO recommendations to define individuals with a healthy weight (BMI 20–25), *overweight *(BMI 25–29.9) and *obese *(BMI≥30) [1]. Individuals with a BMI ≤20 kg/m^2 ^were classified as underweight. Waist circumference (WC), taken midway between the lowest rib and the iliac crest, and hip circumference at the level of the greater trochanters was measured to the nearest millimetre using a flexible tape. Men with a waist circumference of <94, 94–101.9 and ≥ 102 cm were classified as normal weight, overweight and obese respectively, while women were classified in the same obesity categories on the basis of WC <80, 80–87.9 and ≥ 88 cm. Waist-to-hip ratios (WHR) were obtained by dividing waist circumference by hip circumference. Men with WHR < 0.90, 0.90–0.99 and ≥ 1.0 were classified as normal weight, overweight or obese respectively, while women were classified in the same categories on the basis of WHR of < 0.80, 0.80 – 0.84 and ≥ 0.85 [1,16].

### Sociodemographic factors

Sociodemographic factors were assessed using the WHO STEPS instrument. Due to the difficulties in assessing income and profession in the Cameroonian context, the level of education (number of years of schooling, excluding pre-school), was used as the main sociodemographic indicator. The study population was categorised into less than 7 years schooling, 7–14 years and >14 years to correspond to primary, secondary and university levels.

### Data analysis

All analyses were conducted using STATA version 6.0 software [17] and were performed separately for men and women. All analyses were adjusted for the complex sampling design. Age-adjusted partial correlation coefficients were calculated to investigate the association between anthropometric variables. Odd ratios were calculated using logistic regression and are presented with 95% confidence intervals. Statistical significance was set at P < 0.05 (two-tailed) where relevant.

### Ethical issues

All participants gave their written informed consent to participate. The protocol for the Cameroon Burden of Diabetes Baseline Survey was approved by the Cameroon National Ethics Committee.

## Results

The mean age of respondents, estimated taking the stratified sampling design into consideration did not differ significantly between men (31.6 years, 95% CI: 30.8–32.5) and women (31.1 95% CI: 30.5–31.8).

### Anthropometric measurements

Anthropometric measures were obtained for 94%, 94% and 93% of male participants and for 96%, 93% and 95% of women for weight, waist circumference and height respectively. Table 1 shows gender specific means for anthropometric measurements by age group. Men were generally about 10 cm taller than women and the difference was constant across age ranges. Mean levels of BMI and WC, but not WHR, were higher among women than men in all age groups. All anthropometric measurements except height in women peaked within the 45–54 year old age group, and then declined thereafter.

### Correlation between anthropometrics measurements

**Figure 1:**
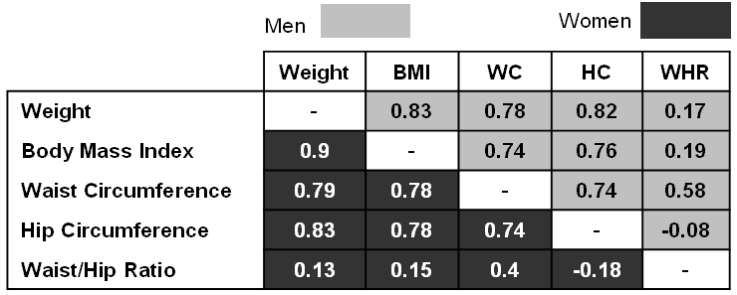
**Age-adjusted partial correlation coefficient among anthropometric measures in urban adults of Cameroon. Findings of the 2003 Cameroon Burden of Diabetes Baseline Survey**. All coefficients significantly different to zero (P < 0.05). WC (Waist Circumference in cm), HC (Hip Circumference in cm), BMI (Body Mass Index in kg/m^2^), WHR (Waist-to-Hip Ratio).

Pair-wise partial correlation between weight, BMI, WC, HC and WHR was investigated, after controlling for age. The results are presented in figure 1. Weight, BMI, WC and HC were generally strongly correlated in both sexes; suggesting that measures of obesity based on these parameters will provide comparable information. However, WHR showed a weaker correlation with the other anthropometric measurements.

### Prevalence of underweight, overweight and obesity

The prevalence of overweight and obesity, based on BMI, WC and WHR are shown in table 2. Irrespective of age or measure used, women always had a higher prevalence of overweight and obesity than men. Based on BMI, over 25% of men and almost half of women were either overweight or obese with 6.5% of men and 19.5% of women in the obese category. The difference in prevalence of obesity between men and women was particularly large in the younger age group (15 – 34 years), where the ratio of prevalence of obese women to obese men was over five (11.8% versus 2.4%).

The prevalence of overweight and obesity varied greatly with age, generally increasing, irrespective of the measurement used. The prevalence of BMI obesity increased with age until 45–54 yrs and then declined (p value for trend < 0.001); this trend was more pronounced among women. There was a particularly large increase between the 15–34 yrs and 35–44 yrs age groups. Moving from the baseline age group category (15–34 yrs) to the next (35–44 yrs) increased the prevalence of obesity as estimated by BMI, WC and WHR by 5.8, 14, and 4.5 fold respectively in men and by almost 3, 3 and 2 times respectively in women.

**Figure 2:**
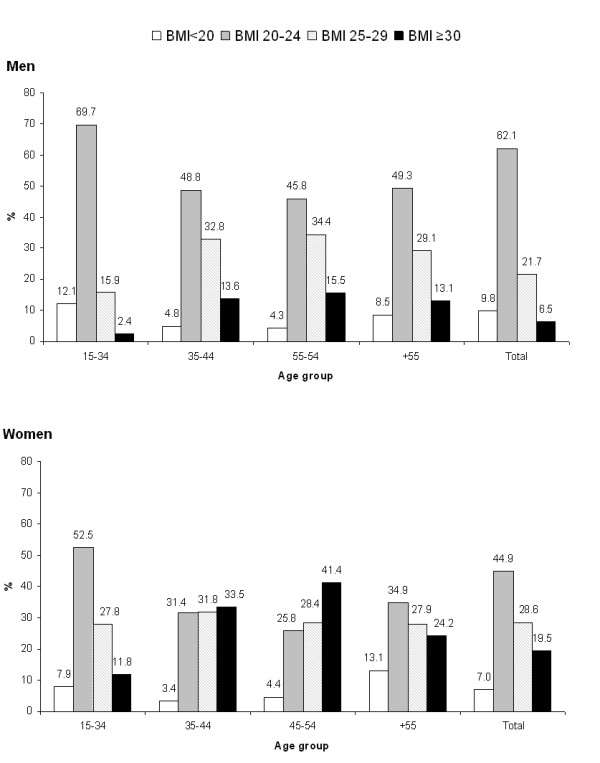
**Distribution of body mass index categories by age group in men and women adults of urban Cameroon. Findings of the 2003 Cameroon Burden of Diabetes Baseline Survey**. BMI (Body mass index, in kg/m^2^).

The prevalence of underweight (BMI<20 kg/m^2^) demonstrated a very different pattern (figure 2) with the highest proportion of underweight in the two extreme age groups (15–34 and 55+ years) in both sexes. The prevalence of underweight was similar between males and females, and was higher than that of obesity in men (9.8% versus 6.5%) but not women. The prevalence of underweight was far less than that of overweight and obesity combined in both sexes, particularly among women (males 9.8% vs 28.1%; women 7.0% vs 48.1%).

### Obesity in relation to age

**Figure 3:**
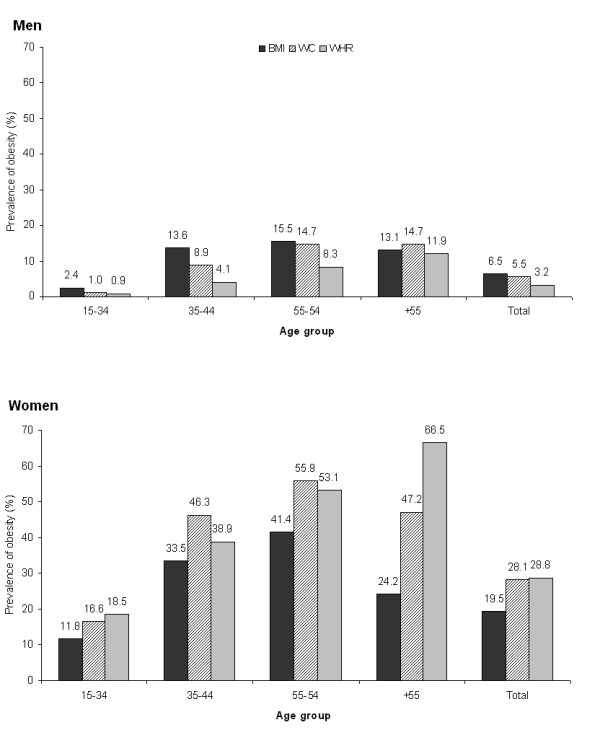
**Prevalence of Obesity as yielded by BMI, WC and WHR, in men and women adults of urban Cameroon. Findings of the 2003 Cameroon Burden of Diabetes Baseline Survey**. BMI (Body Mass Index ≥ 30 kg/m^2^), WC (Waist Circumference ≥ 102 cm in men and ≥ 88 cm in women), WHR (Waist-to-Hip ratio ≥ 1 in men and ≥ 0.85 in women).

Figure 3 shows the distribution of obesity in the study population based on BMI, WC and WHR. The three measurements appear to provide different prevalence of obesity across genders. For example, in the 15–34 years age group WC and WHR give a similar prevalence of obesity in both sexes, but in men BMI provides the highest prevalence estimate, whilst in women it provides the lowest. In the >55 yrs age group WC provided the highest prevalence of obesity in men (though similar to the prevalences using BMI and WHR), while WHR yielded the highest prevalence in women (66.5%), much greater than BMI (24.2%). Overall, using BMI provided the highest prevalence of obesity in men (6.5%) and WHR the lowest (3.2%), while WHR and WC yielded the highest prevalence of obesity in women (28%) and BMI the lowest (19.5%).

### Age-adjusted anthropometric measurements by BMI and WHR categories

The gender specific age-adjusted mean anthropometric measurements of the study participants, according to BMI and WHR categories are displayed in Table 3. The mean values of weight, WC and HC increased markedly in ascending BMI and WHR categories, while WHR showed little change with BMI categories in both genders.

### Obesity and sociodemographic factors

The association between education level and anthropometric measures are shown in table 4. The age-adjusted odd ratio of being overweight or obese or of having a high WC was increased with greater duration of education in both sexes, but particularly strongly in men. In men, having 7–14 years and more than 14 years of education increased the likelihood of overweight or obesity by about 2 and 3.5 fold respectively. In women, this gradient, although present, was less pronounced. WHR was weakly associated with level of education in men, and was weakly negatively associated with level of education in women.

## Discussion

Excess body fat is well documented as being a risk factor for numerous chronic conditions such as diabetes, hypertension, hyperlipidaemia and cardiovascular diseases[18]. Studies of anthropometric measures among adult populations of sub-Saharan Africa countries are limited and weight, and BMI are the most common indicators which have been used to assess overweight and obesity prevalence. The CamBoD Baseline Survey, was the first large scale study of cardiovascular risk factors, using standardized methodology, to be conducted in Cameroon, and the first using the WHO STEPS methodology from Sub-Saharan Africa. This study updates the data on overweight and obesity using a range of anthropometric parameters in the adult population of men and women living in urban area of Cameroon.

This study highlights the high prevalence of overweight and obesity in Cameroon, whether measured by BMI, WC or WHR, and highlights the emergence of non-communicable diseases and their risk factors as major contributors to the burden of ill-health in Sub-Saharan Africa, particularly among urban populations.

The prevalence of obesity estimated from the CamBoD Baseline survey was particularly high in women, and increased markedly between 15–34 and 35–44 years in both sexes. Prevalence of obesity was five times higher in females aged 15–34 years compared to men. Comparison with previous findings in Cameroon is difficult because of different methodologies. However, the prevalence of obesity, estimated from this study is consistent with already published results. Thus, Sobngwi et al. reported a prevalence of BMI obesity of 5.4% in men and 17.1% in women [4] among urban Cameroonians in 2002, slightly lower than found in this study. Pasquet et al [5] also reported comparable results. Lower prevalences have been reported in other urban settings in Africa [19,20], though even higher prevalences were found by Puoane et al who reported a prevalence of 9.2% in men and 42% in women in the urban adult population of South Africa [21].

Obesity as estimated by BMI (a measure of total body fat) and central obesity as estimated by WC and WHR was low among men in the younger age group (0.9–2.4%) and moderate among women (11.8–18.5%). BMI defined obesity increased to 13.6% in 35–44 years old men, and then remained fairly constant in the older age groups, while central obesity measured by WHR continued to increase to a maximum (11.8%) in the >55 years group. BMI defined obesity increased to a peak of 41.4% in the 45–54 years group in women, and increased steadily across all age groups to a maximum of 66.5% in women aged >55 years.

The anthropometric measurements except WHR were strongly correlated with each other. The correlation of indices of overall and central obesity is highly suggestive of an association between increased overall obesity (as measured by BMI) with increased visceral fat (WC in this case). We found that mean WC, more than WHR, increased across overall obesity (BMI) categories in both genders. Similar results were reported in the urban female population of Morocco by Belahsen et al [22], and by Sargeant et al in the urban adult population of Jamaica [23]. It is likely therefore that BMI and WHR provide different measures of almost the same phenomenon.

Visceral fat is more metabolically active than subcutaneous fat, and hence may be more deleterious to health [9]. Several studies have found a strong association between visceral fat and cardiovascular risk factors [24-27]. WC is a practical measure of intra-abdominal fat mass [25] and recommendations have been formulated to use it in the identification of people in need of intervention for cardiovascular risk reduction [28-30]. In men and women adults of urban Cameroon, WC was strongly correlated with BMI (r ≈ 0.8 in men and women respectively) but showed moderate correlation with WHR (0.6 for men and 0.4 for women). These findings suggest that defining obesity on the base of waist circumference may be an equally or more valid and useful method for use in epidemiological research and clinical practice, though further research is needed to demonstrate this unequivocally

Studies in developed countries show an inverse (negative) relationship between education and obesity, particularly among women: the lower the education or the social class, the higher the prevalence of obesity. However, in developing societies, a strong positive relationship often exists between SES and obesity among men, women, and children [31]. In the present paper, the positive association between obesity and duration of education, as measured by the number of year of schooling was clearly seen in both genders when obesity was defined by BMI or WC. The same pattern was less evidence for WHR, particularly in women where there was some evidence of an inverse gradient. Number of years of education can be used as a proxy for socioeconomic status in this population; the greater the number of year of education, the higher the SES. It is likely that higher socioeconomic status in developing countries is characterized by a westernization of the lifestyle, including reduced physical activity, more sedentary life and adoption of higher energy, higher fat diets; all of which may lead to an increase in obesity. However, it is also possible that if westernization of diet and reduced physical activity becomes more general in developing country populations, the familiar inverse association of obesity with socioeconomic status will emerge.

## Conclusion

The 2003 CamBoD Baseline Survey updates the prevalence of obesity in the urban adult population of Cameroon. The findings suggest that waist circumference as a measure of central obesity is a useful indicator for use in epidemiological studies and clinical practice. The study provides insights into the relationship between age, sex, socioeconomic overweight and obesity using a range of anthropometric parameters. Based on BMI alone, the study showed a high prevalence of overweight and obesity and provides evidence to support the establishment of intervention programmes to prevent further increase in obesity-related disorders such as diabetes and hypertension in urban Sub-Saharan African populations. Meanwhile, further studies need to be carried out in the Cameroonian context and other Sub-Saharan African settings to provide appropriate cut-off points and identify which anthropometric parameter has the highest predictive value in the identification of subjects at risk of obesity-related disorders.

## Competing interests

The author(s) declare that they have no competing interests.

## Authors' contributions

JCM, NU, RE, JSA participated in the design of the Cameroon Burden of Diabetes Project study protocol. JSA, RMK and ECK coordinated field data collection. RMK drafted the manuscript and performed all the statistical analysis. All authors provided comments on the draft manuscripts and read and approved the final manuscript.

## Pre-publication history

The pre-publication history for this paper can be accessed here:



## Figures and Tables

**Table 1:** Mean anthropometric (standard error) variables by age groups, in urban adult population of Cameroon. Findings of the 2003 Cameroon Burden of Diabetes Baseline Survey

	***N***	**Height**	**Weight**	**BMI**	**WC**	**HC**	**WHR**
							
**Men**							
15 – 34	2116	170.5 (0.15)	65.5 (0.40)	22.5 (0.11)	75.9 (0.20)	91.4 (0.21)	83.2 (0.20)
35 – 44	558	172.1 (0.48)	74.6 (0.31)	25.1 (0.17)	86.1 (0.72)	97.2 (0.64)	88.5 (0.29)
45 – 54	509	170.8 (0.31)	74.8 (0.37)	25.5 (0.13)	89.0 (0.68)	97.4 (0.67)	91.3 (0.30)
55+	563	168.7 (0.36)	70.1 (0.64)	24.6 (0.19)	87.6 (0.65)	95.0 (0.46)	92.0 (0.35)
Total	3746	170.6 (0.12)	68.3 (0.31)	23.4 (0.09)	80.0 (0.27)	93.3 (0.22)	85.7 (0.18)
**Women**							
15 – 34	3328	161.3 (0.13)	64.1 (0.34)	24.6 (0.13)	78.0 (0.34)	97.6 (0.32)	80.1 (0.29)
35 – 44	1156	162.0 (0.23)	73.4 (0.35)	27.9 (0.17)	87.3 (0.58)	104.9 (0.53)	83.3 (0.33)
45 – 54	631	161.6 (0.25)	75.4 (0.73)	28.9 (0.32)	90.8 (0.75)	106.3 (0.75)	85.9 (0.69)
55+	593	157.5 (0.28)	65.2 (1.02)	26.1 (0.41)	87.7 (1.00)	99.9 (0.99)	88.1 (0.68)
Total	5708	161.3 (0.10)	67.0 (0.28)	25.7 (0.11)	81.7 (0.31)	100.0 (0.27)	81.8 (0.22)

WC (Waist Circumference in cm), HC (Hip Circumference in cm), BMI (Body Mass Index in kg/m^2^), WHR (Waist Hip Ratio).

**Table 2:** Prevalence of obesity by body mass index, waist circumference and waist-hip ratio in adult men and women aged 15 and above. Findings of the 2003 Cameroon Burden of Diabetes Baseline Survey

		**BMI**		**WC**		**WHR**	
				
Age groups	***n***	**Overweight**	**Obese**	**Overweight**	**Obese**	**Overweight**	**Obese**
**Men**							
15 – 34	2116	15.9	2.3	1.7	0.9	5.7	0.8
35 – 44	558	32.8	13.6	13.7	8.9	30.3	4.1
45 – 54	509	34.4	15.5	20.1	14.7	46.2	8.2
55+	563	29.0	13.0	16.7	14.7	47.8	11.9
Total	3746	21.6	6.5	7.5	5.5	17.8	3.2
**Women**							
15 – 34	3328	27.8	11.8	20.3	16.5	25.0	18.5
35 – 44	1156	31.7	33.5	24.0	46.2	25.4	38.9
45 – 54	631	28.3	41.4	20.6	55.8	20.6	53.1
55+	593	27.9	24.2	21.2	47.1	18.7	66.5
Total	5708	28.6	19.5	21.1	28.1	24.3	28.8

**BMI**: Body Mass Index. Overweight was defined as BMI between 25–29.9 and obesity as BMI ≥ 30 kg/m^2 ^in men and women. **WC**: Waist Circumference. Overweight was defined based on WC 94–101.9 cm and 80–87.9 in men and women respectively and obesity based on WC ≥ 102 cm in men and ≥ 88 cm in women. **WHR**: Waist-to-hip ratio was defined based on WHR 0.90–0.99 and WHR 0.80–0.84 in men and women respectively and obesity based on WHR ≥ 1 in men and ≥ 0.85 in women)

**Table 3:** Mean (Standard error) anthropometric measures, by body mass index obesity categories in adult men and women of Cameroon. Findings of the 2003 Cameroon Burden of Diabetes Baseline Survey

	***N***	**Weight**	**WC**	**HC**	**WHR**	**BMI**
**Men**						
Underweight	338	50.8 (0.73)	69.2 (0.60)	82.8 (0.67)	0.83 (0.010)	17.8 (0.09)
Normal Weight	2244	64.8 (0.28)	76.5 (0.18)	90.9 (0.30)	0.84 (0.002)	22.1 (0.13)
Overweight	880	78.5 (0.50)	87.9 (0.70)	99.7 (0.42)	0.88 (0.004)	26.8 (0.07)
Obese	284	94.6 (0.80)	103.0 (0.55)	110.5 (0.45)	0.93 (0.004)	33.7 (0.32)
						
WHR < 0.90	2791	66.1 (0.91)	76.5 (0.58)	92.4 (0.72)	0.82 (0.001)	22.6 (0.35)
WHR ≥ 0.90	1216	76.2 (1.02)	92.7 (0.73)	96.6 (0.81)	0.96 (0.004)	26.3 (0.38)
						
**Women**						
Underweight	401	46.0 (0.50)	67.4 (0.64)	84.9 (0.28)	0.79 (0.008)	17.6 (0.14)
Normal Weight	2436	58.0 (0.31)	74.5 (0.35)	93.4 (0.30)	0.80 (0.001)	22.2 (0.16)
Overweight	1655	70.7 (0.31)	84.6 (0.39)	103.3 (0.33)	0.82 (0.003)	27.2 (0.05)
Obese	1216	90.0 (0.60)	99.2 (0.43)	115.8 (0.53)	0.86 (0.006)	34.8 (0.13)
						
WHR < 0.85	3792	64.6 (1.03)	77.5 (0.77)	99.3 (0.90)	0.78 (0.002)	24.7 (0.42)
WHR ≥ 0.85	2212	72.5 (1.23)	92.6 (0.84)	101.6 (0.77)	0.91 (0.005)	28.0 (0.50)

WC (Waist Circumference in cm), HC (Hip Circumference in cm), BMI (Body Mass Index in kg/m^2^), WHR (Waist Hip Ratio), SE (Standard Error). BMI Categories: Normal Weight (BMI<25 Kg/m^2^), Overweight (BMI 25–29.9 Kg/m^2^), Obese (BMI≥30 Kg/m^2^)

**Table 4:** Age adjusted odd ratios (95% confidence interval) for being overweight or obese based on body mass index, waist circumference and waist-to-hip ratio, with duration of education in men and women adults of urban Cameroon. Findings of the 2003 Cameroon Burden of Diabetes Baseline Survey

	**Men**				**Women**			
	
Duration of Education	n	BMI≥25	WC≥0.90	WHR≥0.95	n	BMI≥25	WC≥0.80	WHR≥0.85
<7 Years	732	Reference	Reference	Reference	1704	Reference	Reference	Reference
7–14 years	1779	1.94 (1.34 – 2.85)	2.31 (1.65 – 3.25)	1.38 (0.90 – 2.12)	2737	1.87 (1.44 – 2.42)	1.52 (1.19 – 1.93)	0.86 (0.71 – 1.04)
≥14 Years	1292	3.41 (2.15 – 5.41)	4.73 (3.17 – 7.00)	1.83 (1.23 – 2.74)	1235	2.23 (1.72 – 2.90)	1.88 (1.52 – 2.33)	0.69 (0.55 – 0.87)

**BMI**: Body Mass Index. Overweight was defined as BMI between 25–29.9 and obesity as BMI ≥ 30 kg/m^2 ^in men and women. **WC**: Waist Circumference. Overweight was defined based on WC 94–101.9 cm and 80–87.9 in men and women respectively and obesity based on WC ≥ 102 cm in men and ≥ 88 cm in women. **WHR**: Waist-to-hip ratio was defined based on WHR 0.90–0.99 and WHR 0.80–0.84 in men and women respectively and obesity based on WHR ≥ 1 in men and ≥ 0.85 in women.
